# IDH1 Mutations in Acute Myeloid Leukemia: Frequency and Clinical Features in the Context of FLT3 and NPM1 Co-Mutations—A Single-Center Study from Turkey

**DOI:** 10.3390/cimb48080752

**Published:** 2026-07-24

**Authors:** Yunus Catma, Simge Erdem, Aynur Aday, Aysegul Bayrak Tokac, Metban Guzel Mastanzade, Murat Ozbalak, Ipek Yonal Hindilerden, Mustafa Nuri Yenerel, Meliha Nalcaci, Sukru Ozturk, Sevgi Kalayoglu Besisik

**Affiliations:** 1 Faculty of Medicine, Department of Internal Medicine, Division of Hematology, Istanbul University, 34093 Istanbul, Turkey; simge_85@hotmail.com (S.E.); metban@gmail.com (M.G.M.); mozbalak@istanbul.edu.tr (M.O.); ipekyonal@hotmail.com (I.Y.H.); mnyenerel@gmail.com (M.N.Y.); mnalcaci@hotmail.com (M.N.); sevgikalayoglu@gmail.com (S.K.B.); 2 Faculty of Medicine, Department of Internal Medicine, Division of Genetics, Istanbul University, 34093 Istanbul, Turkey; aynur.aday@istanbul.edu.tr (A.A.); abayrak@istanbul.edu.tr (A.B.T.); sozturk@istanbul.edu.tr (S.O.)

**Keywords:** acute myeloid leukemia (AML), isocitrate dehydrogenase 1 (IDH1) mutation, molecular characterization

## Abstract

The prognostic relevance of IDH1 mutations remains unclear in acute myeloid leukemia (AML). This study, representing the first Turkish AML cohort characterized for IDH1 mutations, aimed to determine the frequency of IDH1, FLT3 and NPM1 mutations and to describe the associated clinical characteristics. We retrospectively analyzed clinical, cytogenetic, and molecular data for 57 AML patients. Bone marrow aspirates were evaluated cytogenetically using G-banding, and molecular mutations were assessed by PCR or NGS (MiSeq-Illumina). IDH1 mutations were detected in 7% of patients; FLT3 and NPM1 mutations were detected in 38.6% and 19.3% of patients, respectively. A significant positive correlation existed between IDH1 and NPM1 mutations (r = 0.388, *p* = 0.003). Overall mortality was 56.1% and remission was achieved in 43.9%. Older age (*p* = 0.029) and comorbidities (*p* = 0.009) were significantly linked to mortality. Mortality was 100% in the four IDH1-mutant patients and significantly higher in NPM1-mutant patients. Overall survival was shorter in IDH1-mutants, although the difference did not reach statistical significance (log-rank *p* = 0.057). Relapse occurred in 26.3%, rising to 50% in the IDH1-mutated subgroup. In this limited cohort, IDH1-mutant cases were associated with shorter overall survival and higher mortality; however, the small number of IDH1-mutant patients (*n* = 4) precludes definitive prognostic conclusions. Outcomes in NPM1-mutated patients were also less favorable than typically expected, possibly related to co-occurring IDH1 mutations. These descriptive findings characterize the molecular landscape of AML in an underrepresented Turkish population and may provide a foundation for larger, multicenter studies rather than confirming a prognostic role for these mutations.

## 1. Introduction

Acute myeloid leukemia (AML) is a genetically diverse hematologic malignancy, with cytogenetic abnormalities observed in 50–60% of newly diagnosed cases, including balanced translocations, unbalanced alterations, and complex karyotypes [1]. The 2022 WHO and ICC classifications introduced “AML with defining genetic abnormalities,” encompassing key mutations such as NPM1, biallelic CEBPA, RUNX1, TP53, and KMT2A rearrangements [2,3].

In addition, mutations in genes such as FLT3, NPM1, and IDH1/2 have been extensively studied for their prognostic significance. The isocitrate dehydrogenase (IDH) gene mutations, particularly in IDH1, contribute to leukemogenesis through the production of the oncometabolite 2-hydroxyglutarate, which disrupts normal cellular differentiation, induces DNA hypermethylation, and alters epigenetic regulation [4]. IDH mutations are also reported in various malignancies, including gliomas, cholangiocarcinomas, and chondrosarcomas, highlighting their broader oncogenic potential [5]. IDH-mutated AML frequently presents with a normal karyotype. The prognostic impact of IDH1 mutations in AML appears to be context-dependent, with several studies reporting adverse clinical outcomes in the general AML population, while others have demonstrated improved survival following allogeneic hematopoietic stem cell transplantation in selected patient groups [6].

Across published cohorts the genes examined here show characteristic mutation frequencies in AML. IDH1 mutations, most often the R132 variant, have been reported in approximately 3–20% of patients: ElNahass et al. found 2.9% among 70 de novo cases [7], Maggioni et al. 10–20% in 477 patients [8], Paschka et al. 7.6% in 805 adults [9], and Chotirat et al. 8.7% [10]. NPM1 mutations occur in about 30% of newly diagnosed cases, particularly with normal cytogenetics, and FLT3 mutations in 25–35%, with FLT3-ITD reported in 24% of the 177-patient cohort of Garciaz et al. [11,12]. In the cohort of Middeke et al., 52% of patients had a normal karyotype [13]. IDH1 mutations co-occur with NPM1 mutations in a substantial proportion of IDH-mutant cases, reaching 60–74% in some series, and IDH2 mutations may precede NPM1 during leukemogenesis [9,10,14,15].

Several large cohorts have examined how these mutations influence outcome. A 2024 meta-analysis of 20 studies by Qin et al. found that IDH1 mutations increase mortality risk (HR = 1.62, 95% CI 1.42–1.86) whereas IDH2 mutations do not [16]. In 3898 patients, Middeke et al. reported lower complete remission rates for the R132C variant than for R132H (53% vs. 72%, *p* ≤ 0.001) and a trend toward shorter survival [13], and ElNahass et al. described higher blast percentages and inferior outcomes in IDH1-mutated patients [7]. NPM1 mutations are conventionally favorable in the absence of FLT3-ITD, yet this advantage may not be uniform: concurrent IDH1 mutations have been linked to induction resistance and higher mortality, and secondary AML-type co-mutations to inferior survival despite favorable-risk classification [14,17]. Older age and comorbidity burden are further well-established determinants of outcome [18].

The therapeutic landscape has evolved with the introduction of targeted IDH inhibitors such as ivosidenib (IDH1 inhibitor) and enasidenib (IDH2 inhibitor), which promote leukemic cell differentiation and have shown promising clinical efficacy in AML. However, resistance mechanisms and relapse following IDH inhibition have also been documented [19]. Although the frequency of IDH1 mutations has been explored in Turkish patients with myeloproliferative neoplasms, with a total variant homozygous rate of 2.3% and subtype-specific frequencies such as 5% in essential thrombocythemia and 1.7% in primary myelofibrosis, there is still a notable lack of studies directly investigating the prevalence of IDH1 mutations in Turkish AML cohorts [20]. To our knowledge, this is the first molecularly characterized AML cohort from Turkey to specifically examine IDH1 mutations. Therefore, we aimed to determine the frequency of IDH1, FLT3 and NPM1 mutations in AML patients within a single-center cohort and to describe the clinical characteristics associated with them.

## 2. Materials and Methods

This single-center, retrospective study was performed with the Institutional Review Board protocol approval date 30 April 2024 and number 2541283 between February 2013 and October 2023. The research protocol adhered to the ethical guidelines of the World Medical Association’s Declaration of Helsinki. Adult patients diagnosed with AML during this period who had available clinical, cytogenetic, and molecular data at the time of diagnosis were included, yielding a total of 57 patients. Diagnostic classification and risk stratification of AML cases were performed retrospectively based on the 2017 European LeukemiaNet (ELN) recommendations, as these represented the applicable guidelines at the time of initial diagnosis and treatment. Patient demographics, clinical presentations, laboratory findings, cytogenetic and molecular results from bone marrow aspirates at admission, treatment details, and outcomes were obtained from the hospital’s electronic medical records system.

Upon admission, all patients underwent comprehensive laboratory testing, including complete blood count (CBC), lactate dehydrogenase (LDH), prothrombin time (PT), activated partial thromboplastin time (aPTT), D-dimer, C-reactive protein (CRP), and creatinine levels.

### 2.1. Molecular and Cytogenetic Analysis

Bone marrow aspirate samples were cultured for 24 h without stimulation, following standard protocols for cytogenetic analysis. The cultured cells underwent G-banding, and metaphase chromosomes were analyzed using light microscopy to identify chromosomal abnormalities. At least 20 metaphases were examined per sample to ensure accurate karyotyping.

FLT3 and NPM1 Mutations: Data on FLT3 internal tandem duplications (ITD), tyrosine kinase domain (TKD) mutations, and NPM1A mutations were retrieved from patient records. These mutations had been previously assessed using polymerase chain reaction (PCR)-based methods on bone marrow aspirate samples. Additionally, DNA was extracted from bone marrow aspirate samples collected at diagnosis and stored appropriately. The IDH1 mutation analysis was performed using next-generation sequencing (NGS) on an Illumina platform at the Intergen Genetic Diseases Evaluation Center. This method allows for the detection of mutations with high sensitivity and specificity.

### 2.2. Statistical Analysis

All data were analyzed with SPSS (Statistical Package for the Social Sciences) software for Windows (v21.0; IBM, Armonk, NY, USA). Individual and aggregate data were summarized using descriptive statistics including mean, standard deviations, medians (min-max), frequency distributions and percentages. Normality of data distribution was verified by the Kolmogorov–Smirnov test. Comparison of the variables with normal distribution was made with Student t test. For variables that were not normally distributed, the Mann–Whitney U and Kruskal–Wallis tests were used for between-group comparisons. Evaluation of categorical variables was performed by Chi-Square test. Fisher’s exact test was used for categorical comparisons in which expected cell counts were small. Survival outcomes were evaluated using the Kaplan–Meier method, presented as survival curves, and compared between groups using the log-rank test. *p*-values of < 0.05 were considered statistically significant.

## 3. Results

The mean age of the patient cohort was 46.05 ± 15.19 years (range: 20–77), with a balanced gender distribution (52.6% male, *n* = 30; 47.4% female, *n* = 27). At least one comorbidity was present in 17 patients (29.8%), including diabetes (*n* = 6), hypertension (*n* = 8), coronary artery disease (*n* = 3), and solid tumors (*n* = 2). Glomerular filtration rate (GFR) was below 60 mL/min in 4 patients (7%), and 7 patients (12%) presented with disseminated intravascular coagulation (DIC) at diagnosis.

**Figure 1:**
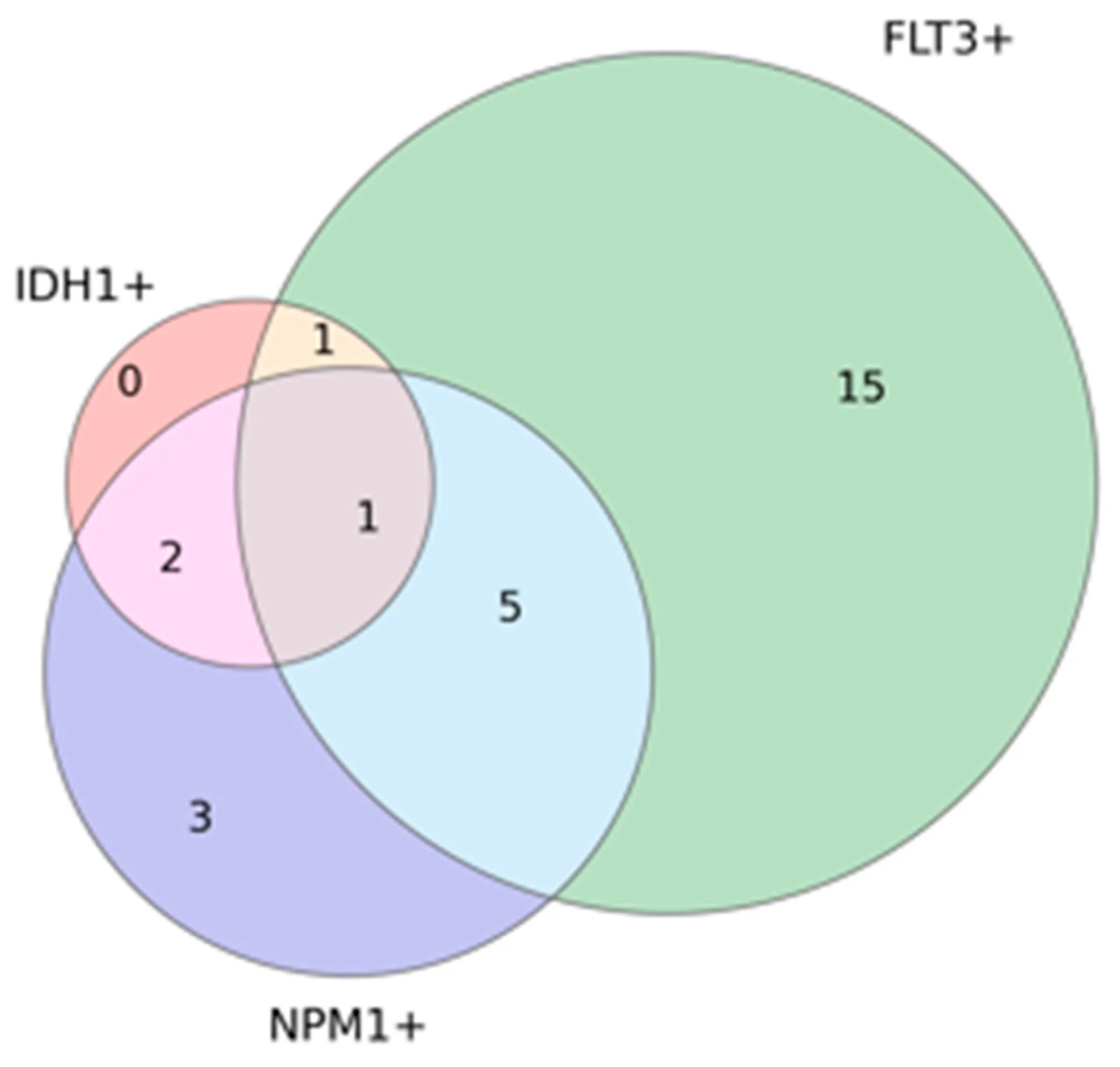
Venn diagram showing the overlap of IDH1, FLT3, and NPM1 mutations in AML patients.

Molecular analysis revealed IDH1 mutations in 7.0% (*n* = 4) of cases, while FLT3 mutations were identified in 38.6% (*n* = 22), including FLT3-ITD in 29.8% (*n* = 17) and FLT3-TKD in 8.8% (*n* = 5). NPM1 mutations were detected in 19.3% (*n* = 11) of patients. All four IDH1-mutant patients carried at least one co-occurring mutation. In addition, 18 patients (31.6%) exhibited isolated single-gene mutations, including FLT3-TKD in 4 patients (7.0%), FLT3-ITD in 11 patients (19.3%), and NPM1 in 3 patients (5.3%). Eight patients (14.0%) harbored two concurrent mutations: IDH1 + NPM1 in 2 patients (3.5%), NPM1 + FLT3-ITD in 5 patients (8.8%), and FLT3-ITD + IDH1 in 1 patient (1.8%). A single patient (1.8%) demonstrated triple co-mutation with concurrent NPM1, IDH1, and FLT3-TKD mutations (Table 1) (Figure 1).

Using NGS analysis, the IDH1-R132H mutation was detected in four patients. The variant allele frequency (VAF) for the IDH1-R132H mutation was 50% in two patients, 15% in one patient, and 7% in the other. No other types of IDH1 mutations were detected. Cytogenetic abnormalities were detected in 40.8% (*n* = 20/49) of patients at diagnosis. In addition, a statistically significant positive correlation was found between IDH1 and NPM1 mutations (r = 0.388, *p* = 0.003). Allogeneic hematopoietic stem cell transplantation (allo-HSCT) was performed in 50.9% (*n* = 29) of patients.

Treatment regimens varied among patients but largely followed established AML protocols. The most common induction regimen was the standard “3 + 7” protocol, comprising cytarabine (ARA-C) for 7 days and an anthracycline (idarubicin or daunorubicin) for 3 days, administered to 70.2% (*n* = 40) of patients. Other induction therapies included ATRA plus idarubicin in 14.0% (*n* = 8), low-dose azacitidine (AZA) alone in 3.5% (*n* = 2), AZA combined with venetoclax in 1.8% (*n* = 1), and hydroxyurea-based regimens in 3.5% (*n* = 2). A small subset also received ARA-C monotherapy (3.5%, *n* = 2). For re-induction, FLAG-IDA (fludarabine, cytarabine, G-CSF, and idarubicin) was administered in 15.8% (*n* = 9) of patients. Regarding consolidation therapy, high-dose cytarabine (HiDAC) was the most frequently used regimen, given to 64.9% (*n* = 37), with 7.0% (*n* = 4) receiving HiDAC combined with ATRA and idarubicin. Overall, 28.1% (*n* = 16) of patients did not receive consolidation therapy, mostly due to early mortality or comorbidities.

At last follow-up, 56.1% (*n* = 32) of patients had died and 43.9% (*n* = 25) were alive in remission. Considered separately, first complete remission was achieved in 66.7% (*n* = 38) of patients, whereas 33.3% (*n* = 19) did not attain remission; baseline clinical and molecular characteristics were evaluated in relation to this first complete remission status. No statistically significant differences were observed between these groups in terms of gender (male: 73.3% vs. female: 59.3%, *p* = 0.260), comorbidity status (no comorbidity: 72.5% vs. comorbidity present: 52.9%, *p* = 0.152), mutation status for IDH1 (remission in wild-type: 66.0% vs. mutant: 75.0%, *p* = 0.714), FLT3 (wild-type: 62.9% vs. mutant: 72.7%, *p* = 0.442), and NPM1 (wild-type: 69.6% vs. mutant: 54.5%, *p* = 0.342) or cytogenetic abnormalities (abnormal: 60.0% vs. normal: 75.9%, *p* = 0.236). In addition, mortality and remission groups were compared based on clinical characteristics (Table 2). Patients who succumbed to the disease had a significantly higher mean age at diagnosis (49.91 ± 14.45 years) compared to those in remission (41.12 ± 14.97 years, *p* = 0.029). Additionally, patients with at least one comorbidity had a significantly higher mortality rate than those without (82.4%, 14/17 vs. 45.0%, 18/40; *p* = 0.009). All four patients with IDH1 mutations had fatal outcomes, whereas the mortality rate in the IDH1 wild-type group was 52.8% (*p* = 0.067). Furthermore, the mortality rate was significantly higher in NPM1-mutated patients (90.9%) compared to those without NPM1 mutations (47.8%, *p* = 0.01). When patients were grouped based on mutational status as single, double, or triple mutations, no statistically significant association was observed with final clinical outcomes (remission vs. exitus) (*p* = 0.114). Among patients with single mutations (*n* = 18, 31.6%), 55.6% (*n* = 10) achieved remission. For those with two concurrent mutations (*n* = 8, 14.0%), only 12.5% (*n* = 1) reached remission, while 87.5% (*n* = 7) were deceased. The patient with a triple mutation (IDH1 + NPM1 + FLT3-TKD) (1.8%, *n* = 1) experienced a fatal outcome. She was a 54-year-old female who was followed for 14 months and ultimately died due to sepsis and pulmonary embolism. Patients with no mutation (*n* = 30, 52.6%) had a remission rate of 46.7% (*n* = 14). Patients who underwent allo-HSCT had significantly lower mortality rates (41.4% vs. 71.4%, *p* = 0.022) compared to those who did not receive transplantation (Table 2).

**Figure 2:**
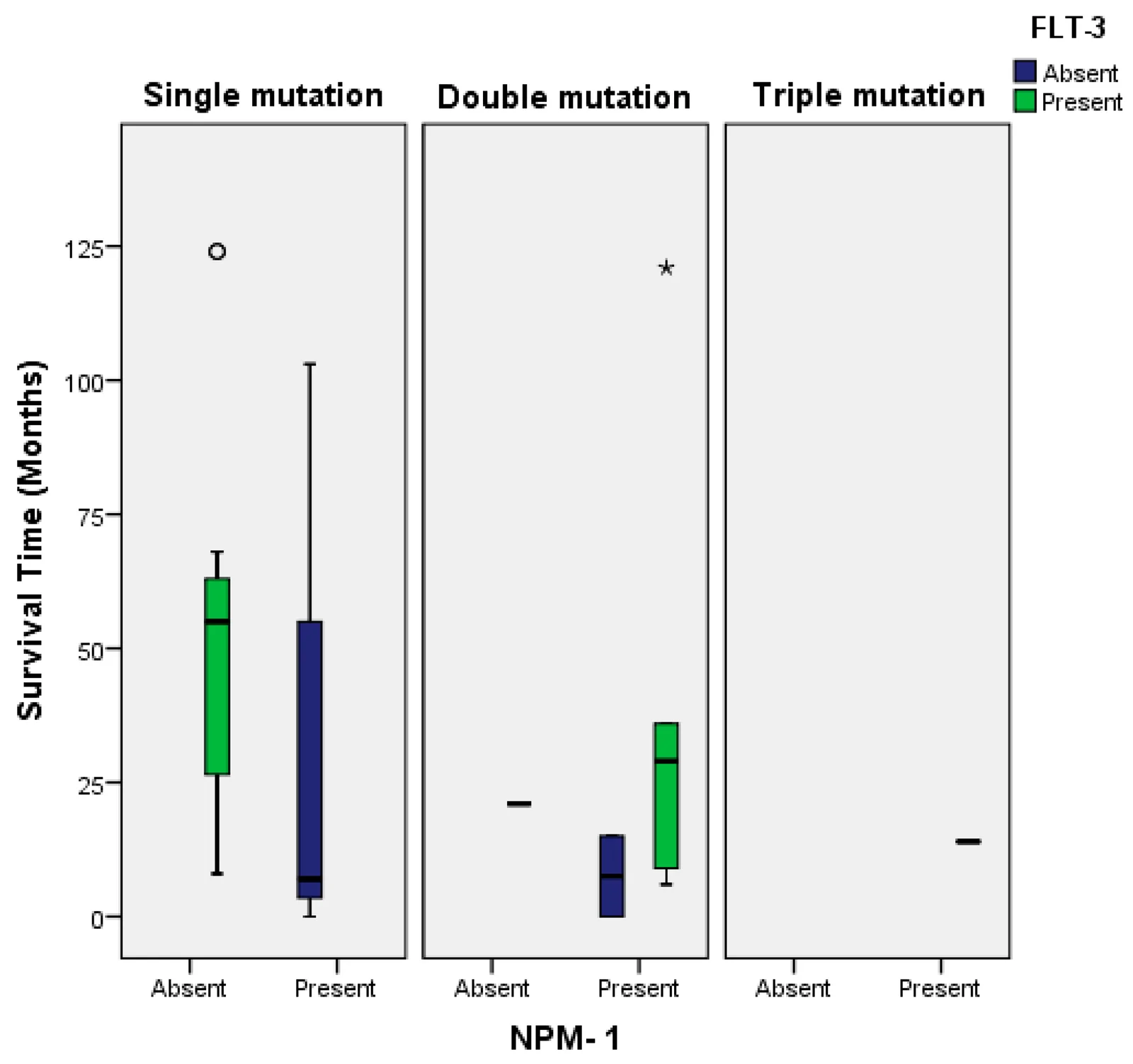
Mean overall survival times according to mutation status.

**Figure 3:**
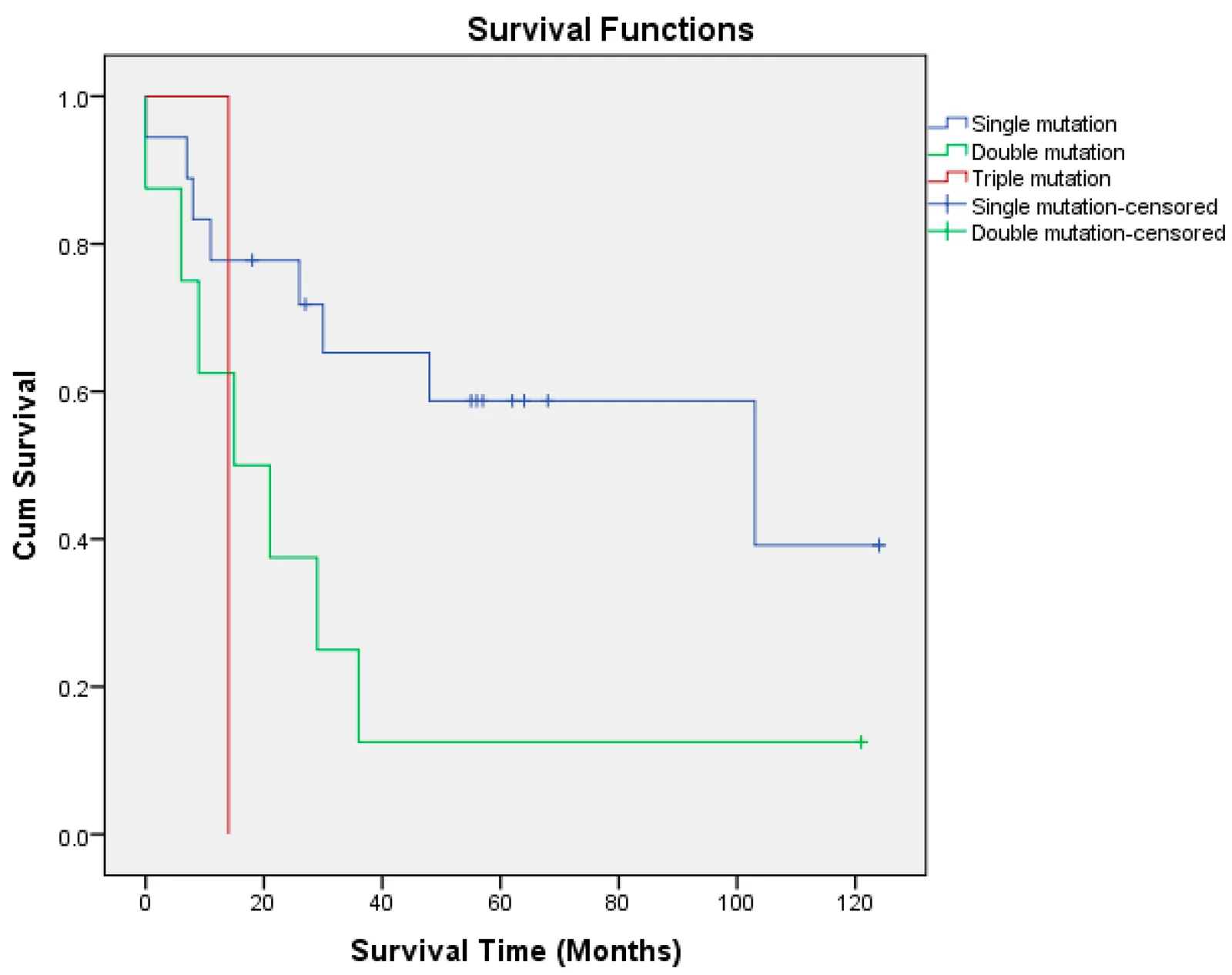
Kaplan–Meier overall survival curves according to mutation status (log-rank *p* = 0.057).

**Figure 4:**
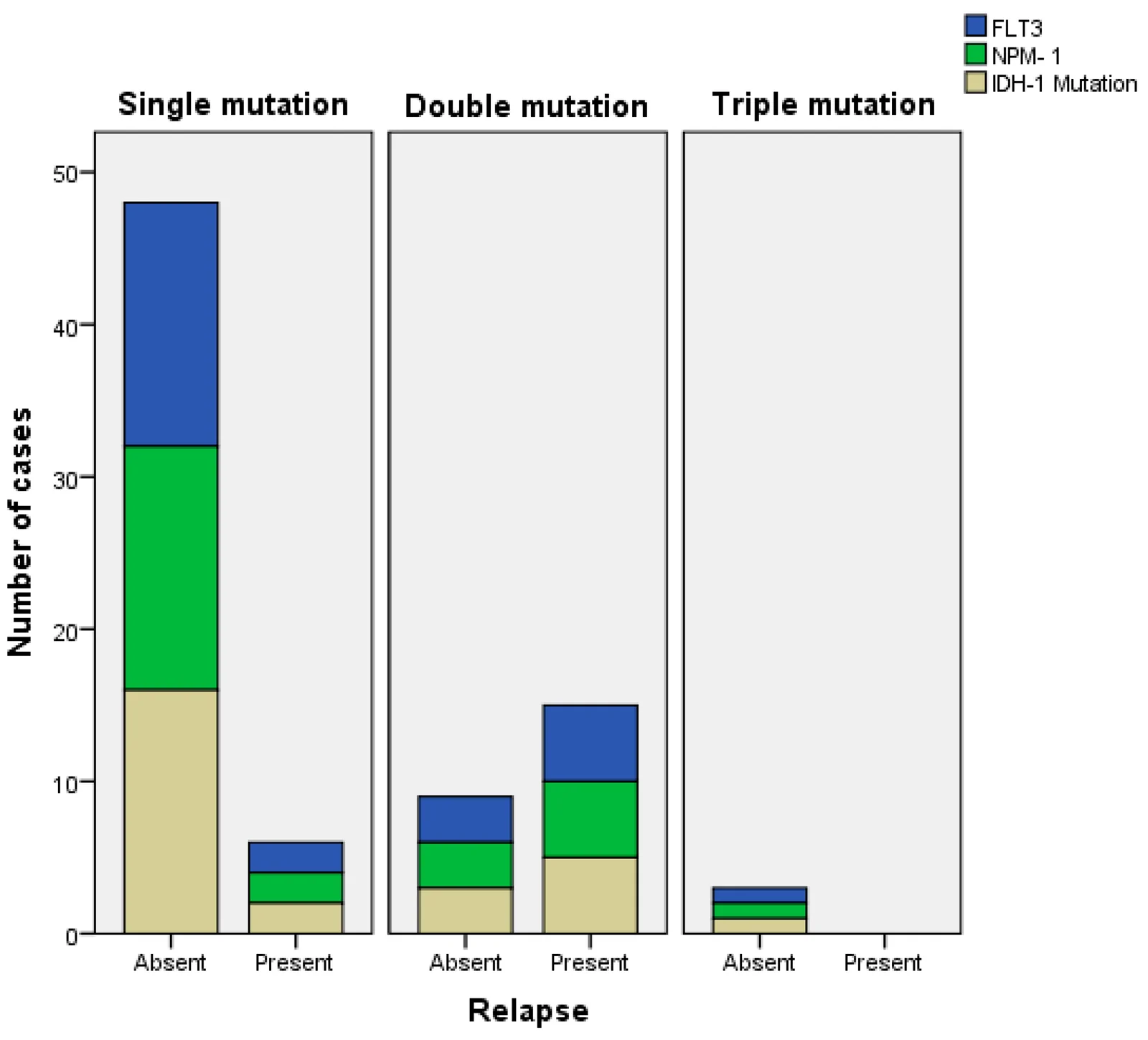
Comparison of relapse rates according to mutation status.

The mean overall survival (OS) was 39.51 ± 38.91 months (range: 0–135 months) in present study. Moreover, the mean OS was 12.50 ± 8.88 months in IDH1-mutant patients, 46.09 ± 36.93 months in FLT3-mutant patients, and 30.91 ± 41.79 months in NPM1-mutant patients (*p* = 0.384) (Figure 2). The mean OS was lower in patients with IDH1 mutations (12.50 ± 8.88 months) than in IDH1 wild-type patients (41.55 ± 39.57 months); on Kaplan–Meier analysis, however, this difference did not reach statistical significance (log-rank *p* = 0.057), indicating a non-significant trend toward shorter survival in the IDH1-mutant subgroup (Figure 3). A relapse was observed in 15 patients (26.3%), with a relapse rate of 50.0% (2/4) in IDH1-mutant cases, 22.7% (5/22) in FLT3-mutant cases, and 45.5% (5/11) in NPM1-mutant cases. However, when compared to mutation-negative patients, no statistically significant association was found between mutation status and relapse risk (*p* > 0.05) (Figure 4). The mean time to relapse was approximately 11.69 ± 7.53 months in IDH1-mutant patients and 10.50 ± 6.36 months in IDH1 wild-type patients (*p* = 0.843).

## 4. Discussion

The principal constraint of this study must be stated at the outset. Although the cohort comprised 57 patients, IDH1 mutations were identified in only four. Survival and relapse estimates derived from a subgroup of this size cannot support prognostic conclusions, and this limitation is compounded by the heterogeneity of treatment regimens across a ten-year period. The observations below are therefore descriptive and hypothesis-generating, and are not intended to establish the prognostic value of IDH1 mutations.

The mutation frequencies in our cohort were broadly consistent with published data. IDH1 mutations were detected in 7% of patients, within the 3–20% range reported across cohorts [7,8,9,10]. FLT3 mutations were present in 38.6%, somewhat above the commonly cited 25–35%, whereas NPM1 mutations were found in 19.3%, below the approximately 30% reported in large series [11,12]. Cytogenetic abnormalities were present in 40.8%, somewhat below the 50–60% commonly reported [1] and close to the proportion implied by the cohort of Middeke et al. [13]. The lower NPM1 frequency may reflect the limited cohort size or population-specific features. We also observed a statistically significant positive correlation between IDH1 and NPM1 mutations (r = 0.388, *p* = 0.003), an association well documented previously [9,10,15]; Abd El-Lateef et al. reported concurrent NPM1 mutations in five of six IDH1-mutant patients (*p* = 0.008) [14].

Two findings concerning established prognostic determinants were consistent with the literature. Patients who died were older at diagnosis (49.91 vs. 41.12 years) and those with at least one comorbidity had higher mortality (82.4% vs. 45.0%), in keeping with the large analysis of Dhakal et al., in which age above 60 years and a Charlson Comorbidity Index of 2 or more were independently associated with greater early mortality and reduced survival [18]. Mortality was also lower among patients who underwent allogeneic hematopoietic stem cell transplantation (41.4% vs. 71.4%, *p* = 0.022), consistent with the reduction in non-relapse mortality reported by Grosicki et al. in 622 patients in first complete remission [21] and with the predictive value of the Pretransplant Assessment of Mortality score validated by Middeke et al. [22].

Within the IDH1-mutant subgroup, all four patients died during follow-up and relapse occurred in 50.0%; mean overall survival was lower than in wild-type patients, although this difference did not reach statistical significance on log-rank analysis (*p* = 0.057). The direction of these observations echoes previous reports: Paschka et al. [9] found IDH mutations in NPM1-mutated cytogenetically normal AML associated with worse relapse-free and overall survival, a Mexican cohort reported markedly shorter survival in IDH1-mutant patients [23], and Chin et al. [24], Schnittger et al. [25] and Marvin-Peek et al. [26] each described a substantial relapse burden despite initial response. With four patients, however, these parallels constitute a signal to be tested rather than a finding, and the absence of statistical significance in our data should be given greater weight than the direction of the trend.

Mortality was higher among NPM1-mutated patients (90.9% vs. 47.8%, *p* = 0.01) and their mean overall survival was 30.91 months, which is at variance with the favorable profile usually attributed to NPM1. Co-occurring IDH1 mutations, small subgroup size, and treatment variability are plausible contributors, and the literature is itself divided: Sciumè et al. [27] and Zidan et al. [28] reported heterogeneous outcomes among NPM1-mutated patients, Dunlap et al. [11] found higher relapse and worse survival when IDH1/2 preceded NPM1, whereas Zarnegar-Lumley et al. [29] observed better outcomes with co-occurring IDH and NPM1 mutations and Peterlin et al. [30] found no significant influence of IDH1/2 on relapse risk. Our relapse rates of 50.0% in IDH1-mutated, 45.5% in NPM1-mutated and 22.7% in FLT3-mutated cases showed no statistically significant relationship with mutation status, which is unsurprising given the subgroup sizes.

Data on the molecular and clinical characteristics of AML in Turkish populations remain scarce. A single-center Turkish study of 126 patients reported FLT3-ITD in approximately 25%, t(15;17) in 11%, t(8;21) in 6.3% and inv(16) in 3.7% [31], frequencies broadly comparable to those described in large international cohorts [32,33]. Data on IDH1 mutations in Turkish AML cohorts remain particularly limited, comprising mainly small series and individual case reports [34]. The principal contribution of the present study is therefore descriptive: it characterizes the frequency and clinical context of IDH1, FLT3 and NPM1 mutations in a population underrepresented in global AML genomic data, and provides a reference point for larger, multicenter investigations.

### Limitations

This study has several limitations that should be acknowledged. First, the relatively small sample size (*n* = 57) may limit the generalizability of our findings and restrict the statistical power to perform multivariate analyses. Despite this limitation, we opted to include patients with acute promyelocytic leukemia (APL) due to the already limited cohort size; however, we acknowledge that APL has distinct clinical characteristics and a significantly better prognosis compared to other AML subtypes, which may introduce bias into outcome comparisons. In addition, treatment regimens were heterogeneous across the cohort and spanned a ten-year period; this variability, together with the small subgroup sizes, further limits the interpretability of the survival comparisons, which should therefore be regarded as exploratory rather than confirmatory.

Second, although current ELN guidelines recommend a broader panel of molecular markers for risk stratification in AML, our retrospective dataset—collected over a ten-year period—did not include uniform testing for all ELN-recommended mutations. Our focus remained on IDH1, FLT3, and NPM1 mutations, which were the most consistently reported during this time frame.

These limitations highlight the need for future large-scale, multicenter, and prospective studies to further explore the mutational landscape and its clinical implications in AML, particularly within underrepresented populations such as Turkish cohorts.

## 5. Conclusions

In this single-center cohort, IDH1-mutant AML was associated with a trend toward increased mortality, shorter overall survival, and higher relapse rates. Given the very small number of IDH1-mutant cases (*n* = 4), these observations should be regarded as descriptive and hypothesis-generating rather than confirmatory. The higher mortality observed in NPM1-mutated patients, together with the significant correlation between IDH1 and NPM1 mutations, suggests that the prognostic weight of NPM1 may be context-dependent, but this likewise requires confirmation in adequately powered cohorts. Larger, multicenter studies are needed to clarify the clinical impact of IDH1 and its co-mutations in AML. Nonetheless, this study represents the first analysis of IDH1 mutations in AML conducted in a Turkish cohort and provides preliminary descriptive data as a foundation for future regional research.

## Figures and Tables

**Table 1:** Baseline Clinical and Molecular Characteristics of AML Patients.

Clinical Variables	Value
Mean Age (Mean ± SD) Gender (*n*, %) Female Male	46.05 ± 15.19 years (range: 20–77) 27 (47.4%) 30 (52.6%)
Comorbidities (*n*, %)	17 (29.8%)
Hematologic and Biochemical Parameters (Mean ± SD) WBC count, ×10^3^/µL, median (IQR) Hemoglobin, g/dL, mean ± SD (range) Platelet count, ×10^3^/µL, median (IQR) LDH, U/L, median (IQR) GFR < 60 mL/min, *n* (%) DIC at diagnosis, *n* (%)	16.4 (73) 8.3 ± 2.1 (3–13.9) 46.0 (58) 528 (319) 4 (7%) 7 (12%)
Molecular and Cytogenetic Findings (*n*, %) IDH1 mutation FLT3 mutation FLT3-ITD FLT3-TKD NPM1 mutation Single mutation FLT3-TKD FLT3-ITD NPM1 Double mutation IDH1 + NPM1 NPM1 + FLT3-ITD FLT3-ITD + IDH1 Triple mutation (NPM1 + IDH1 + FLT3-TKD) t(15;17) inv(16) Cytogenetic abnormalities	4 (7%) 22 (38.6%) 17 (29.8%) 5 (8.8%) 11 (19.3%) 18 (31.6%) 4 (7.0%) 11 (19.3%) 3 (5.3%) 8 (14.0%) 2 (3.5%) 5 (8.8%) 1 (1.8%) 1 (1.8%) 5 (8.8%) 2 (3.5%) 20 (40.8%)
Treatment and Outcomes Allogeneic HSCT performed (*n*, %) Total relapse (*n*, %) The overall survival, (Mean ± SD) (range) Overall mortality (*n*, %)	29 (50.9%) 15 (26.3%) 39.51 ± 38.91 months (range: 0–135) 32 (56.1%)

**Table 2:** Comparison of Clinical Characteristics Between Remission and Mortality Groups.

Clinical Variable	Remission *n* (%)	Mortality *n* (%)	*p*-Value
Gender -Male -Female	15 (50.0) 10 (37.0)	15 (50.0) 17 (63.0)	0.325
Comorbidities	3 (17.6)	14 (82.4)	0.009 *
IDH1	0 (0.0)	4 (100.0)	0.067
FLT3/TKD FLT3/ITD	3 (60.0) 8 (47.1)	2 (40.0) 9 (52.9)	0.611
NPM1	1 (9.1)	10 (90.9)	0.01 *
Single mutation Double mutation Triple mutation	10 (55.6) 1 (12.5) 0 (0.0)	8 (44.4) 7 (87.5) 1 (100.0)	0.114
Cytogenetic Abnormality	6 (30.0)	14 (70.0)	0.131
Allogeneic HSCT	17 (58.6)	12 (41.4)	0.022 *

* = *p* < 0.05 statistically significant. Percentages are calculated within each variable category (row-wise).

## Data Availability

The original contributions presented in this study are included in the article. Further inquiries can be directed to the corresponding author.
